# Chest Computed Tomography Is an Efficient Method for Initial Diagnosis of COVID-19: An Observational Study

**DOI:** 10.3389/fmed.2022.848656

**Published:** 2022-04-12

**Authors:** Waldonio de Brito Vieira, Karen Margarete Vieira da Silva Franco, Apio Ricardo Nazareth Dias, Aline Semblano Carreira Falcão, Luiz Fábio Magno Falcão, Juarez Antonio Simões Quaresma, Rita Catarina Medeiros de Sousa

**Affiliations:** ^1^Núcleo de Medicina Tropical, Universidade Federal do Pará, Belém, Brazil; ^2^Centro de Ciências Biológicas e da Saúde, Universidade do Estado do Pará, Belém, Brazil

**Keywords:** COVID-19, severe acute respiratory syndrome, computed tomography, diagnosis, lung injury, CO-RADS

## Abstract

Coronavirus disease (COVID-19) is an infectious disease that can lead to pneumonia, pulmonary oedema, acute respiratory distress syndrome, multiple organ and system dysfunction, and death. This study aimed to verify the efficacy of chest computed tomography (CT) for the initial diagnosis of COVID-19. This observational, retrospective, cross-sectional study included 259 individuals who underwent clinical evaluation, blood collection, chest CT, and a reverse transcription polymerase chain reaction (RT-PCR) diagnostic test for severe acute respiratory syndrome coronavirus 2 (SARS-CoV-2) during their course of treatment at a reference hospital in Belém, Pará, Brazil between April and June 2020. Inclusion criteria were flu-like symptoms in adults of both sexes. Individuals with an inconclusive COVID-19 molecular test or who had artifacts in the chest CT images were excluded. Parametric data were analyzed using Student-*t*-test and non-parametric data were analyzed using average test and Fisher exact test. Participants were divided into two groups: Group 1 (COVID-19 positive), *n* = 211 (124 males, 87 females), 51.8 ± 17.9 years old and Group 2 (COVID-19 negative), *n* = 48 (22 males, 26 females), 47.6 ± 18.6 years old. Most frequent symptoms were cough [Group 1 *n* = 199 (94%)/Group 2 *n* = 46 (95%)], fever [Group 1 *n* = 154 (72%)/Group 2 *n* = 28 (58%)], myalgia [Group 1 *n* = 172 (81%)/Group 2 *n* = 38 (79%)], dyspnoea [Group 1 *n* = 169 (80%) / Group 2 *n* = 37 (77%)], headache [Group 1 *n* = 163 (77%)/Group 2 *n* = 32 (66%)], and anosmia [Group 1 *n* = 154 (73%)/Group 2 *n* = 29 (60%)]. Group 1 had a higher proportion of ground-glass opacity [Group 1 *n* = 175 (83%)/Group 2 *n* = 24 (50%), 0.00], vascular enhancement sign [Group 1 *n* = 128 (60%)/Group 2 *n* = 15 (31%), 0.00], septal thickening [Group 1 *n* = 99 (47%)/Group 2 *n* = 13 (27%), 0.01], crazy-paving pattern [Group 1 *n* = 98 (46%) / Group 2 *n* = 13 (27%), 0.01], consolidations [Group 1 *n* = 92 (43%)/Group 2 *n* = 8 (16%), 0.00], and CO-RADS 4 and 5 [Group 1 *n* = 163 (77.25%)/Group 2 *n* = 24 (50%), 0.00] categories in chest CT. Chest CT, when available, was found to be an efficient method for the initial diagnosis and better management of individuals with COVID-19.

## Introduction

Coronavirus disease (COVID-19) is caused by the severe acute respiratory syndrome coronavirus 2 (SARS-CoV-2) and is characterized by a flu-like syndrome, with the most common initial symptoms being fever, cough, sore throat, fatigue, headache, anosmia, myalgias, and diarrhea (1–6). Although many individuals develop a mild form of the infection and have a good prognosis, COVID-19 can progress to more severe forms with the development of pneumonia, pulmonary oedema, acute respiratory distress syndrome, multiple organ and system dysfunction, and death (7–9). Severe acute respiratory syndrome (SARS) is an important complication in patients with severe disease, and it sets in as soon as individuals progress to dyspnoea and hypoxemia (6).

COVID-19 related-pneumonia is a complication of moderate and severe forms of the disease and are characterized by a higher incidence of bilateral infiltrates, mainly ground-glass opacities and consolidations on chest computed tomography (chest CT) (10, 11). CT scan findings are often used for diagnostic confirmation through protocols such as COVID-19 Reporting and Data System (CO-RADS), which classifies the image findings in CO-RADS categories in accordance with their characteristics, has a good application for triage in symptomatic individuals (12, 13), and helps to monitor the progression of the disease (14, 15).

COVID-19 needs a quick diagnosis, as the severe forms usually have a fast and aggressive progression. The results of the reverse transcriptase polymerase chain reaction (RT-PCR) test, the gold standard, take an average of 7 days to be released by the laboratories, and this time can be the difference between life and death for these patients. Hence, there is a need for a COVID-19 diagnostic method with faster results and good sensitivity.

Chest CT has the potential to quickly deliver a result of imaging patterns characteristic of COVID-19. Hence, this study aimed to verify the efficacy of chest CT for the initial diagnosis of COVID-19.

## Materials and Methods

### Study Design, Ethical Aspects, and Settings

This was a retrospective, cross-sectional, observational study approved by the Research Ethics Committee of Hospital Universitário João de Barros Barreto (Protocol n. 4.010.595). A consent form for data use was obtained from the hospital where the participants were treated. This study was conducted in strict accordance with the principles of the Declaration of Helsinki and was reported in accordance with the Strengthening the Reporting of Observational Studies in Epidemiology (STROBE) guidelines. The study was carried out at a reference hospital in Belém, Pará, Brazil in the Brazilian Eastern Amazon.

### Participants and Materials

All patients of both sexes with flu-like symptoms who underwent investigations including chest CT, blood tests, and nasal swab at the emergency room of the reference hospital were included in this study. Patients with inconclusive results, image artifacts on the chest CT, or incomplete filling of the medical records were excluded. A peripheral arterial oxygen saturation level ≤ 93% was one of the criteria used for hospital admission, according to the institutional protocol.

### Data Collection and Description of the Processes

Symptoms, duration of symptoms, age, sex, peripheral oxygen saturation level at admission, presence of comorbidities, laboratory data, and diagnostic test results of RT-PCR for SARS-CoV-2 were collected from the electronic medical records from TASY^TM^ (Phillips Healthcare^TM^, Amsterdã, Nederlands). All participants underwent a chest CT scan, performed without intravenous contrast in the supine position. Inside the GE Multislice Brightspeed Edge Select CT scanner (GE Healthcare, Chalfont St Giles, UK) using a tube kilovoltage (kV), 100–120 kV; tube current (mAs), automatic exposure control; collimation, 1.0 mm; pitch, 1; reconstruction algorithm, iterative-based reconstruction; reconstruction slice thickness, 0.5 mm; interslice gap, 0 mm and reformatted with lung (width, 1,500 HU; level, −500 HU), and soft tissue (width, 350 HU; level, 50 HU) window settings the patient was instructed to take a deep breath, followed by a momentary apnoea to obtain cross-sectional images of the chest with slices of 1-mm collimation.

The scans were analyzed using the Osirix MD 11.0™ software (Pixmeo Company, Bernex, Suiça) by two radiologists with experience in chest CT, without previous knowledge of the RT-PCR results of the individual patients. Chest tomography analysis was performed according to the qualitative visual assessment of the types of opacities, specifying their morphology, distribution and percentage of involvement of the lung parenchyma, and classification according to the CO-RADS categories.

The chest CT findings were classified as follows: (a) ground-glass opacity, defined as increased density of the lung parenchyma that retains the visible contours of the vessels and bronchi inside the affected area; (b) vascular enhancement sign (VES), vascular enlargement inside the lesion resulting from congestion and dilation of small vessels; (c) septal thickening; (d) crazy-paving pattern appearing as thickened interlobular septa and intralobular lines superimposed on a background of ground-glass opacity; (e) consolidation, when the air in the alveolar space is supplanted by a pathological product; and (f) parenchymal band, appearing as a linear opacity, usually 1–3 mm thick and up to 5 cm long that usually extends to the visceral pleura (16–18).

Chest CT was classified into categories of the Coronavirus disease 2019 Reporting and Data System (CO-RADS). This protocol provides a level of suspicion for pulmonary involvement of COVID-19, based on features seen in the high-resolution chest CT. The level of suspicion gradually increases from CO-RADS 0 to CO-RADS 6 [(12); Table 1].

**Table 1:** The coronavirus disease 2019 reporting and data system (CO-RADS).

**CO-RADS classification**	**Interpretation**
CO-RADS 0	- Non interpretable CT Scan or technically insufficient to determine COVID-19
CO-RADS 1	- Normal CT Scan
CO-RADS 2	- Low compatible with COVID-19 CT Scan
CO-RADS 3	- Equivocal or Unsure COVID-19 CT Scan
CO-RADS 4	- High suspicious for COVID-19 CT Scan
CO-RADS 5	- Very High or typical for COVID-19 CT Scan
CO-RADS 6	- Typical COVID-19 CT Scan with RT-PCR confirmation

The following parameters were also manually measured at CT scans using the Osirix MD 11.0™ software (Pixmeo Company, Bernex, Suiça): diameter of the pulmonary artery trunk whose value when equal to or >29 mm was predictive of pulmonary arterial hypertension (19); dimensions of the left atrium whose hypertrophy was related to systemic arterial hypertension (20); and evaluation of the average density of the hepatic parenchyma, whose densities when <45 Hounsfield Units (HU) suggested hepatic steatosis (21, 22).

According to the results of the RT-PCR tests for SARS-CoV-2, the patients were divided into two groups, Group 1 (COVID-19 positive) and Group 2 (COVID-19 negative), for the purpose of data comparison.

### Statistical Analysis

All the information collected was recorded in spreadsheets of Excel 2007™ software (Microsoft Corporation, Redmond, USA) and analyzed using Graphpad prism 5.0™ (Graphpad software, Inc., San Diego, USA). Lilliefors test was used to assess the normality of the sample. Student *t*-test was used for the analysis of variables with normal distribution, and the average test and Fisher exact test were used for the analysis of the non-parametric variables. The kappa test was used to analyze the interobserver concordance. The α level of 0.05 was adopted to reject the null hypothesis.

## Results

**Figure 1:**
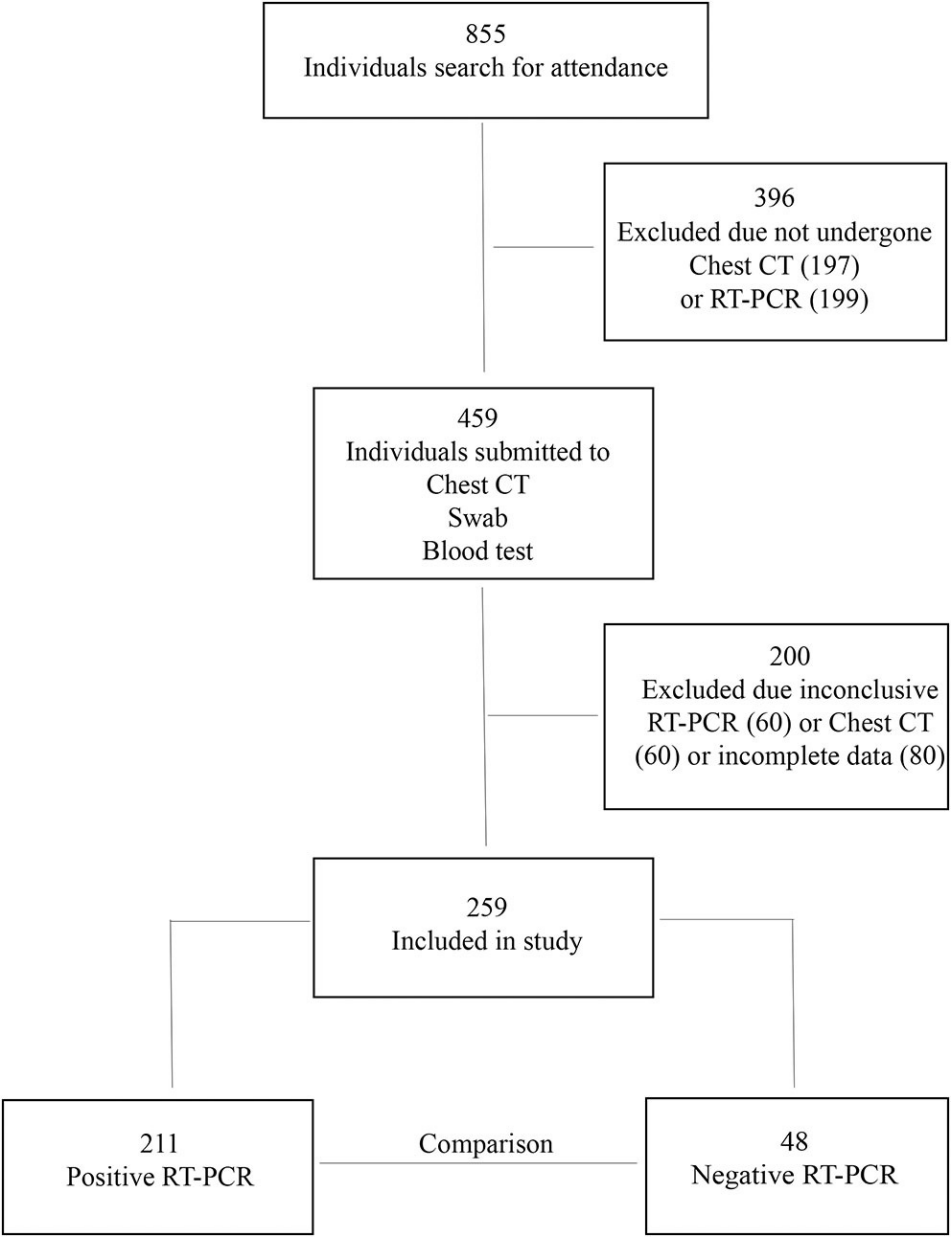
Flowchart of the study.

From 1 April to 30 June 2020, 855 individuals with flu-like symptoms were evaluated (anamnesis and physical examination). Of these, 459 individuals were suspected to have SARS-CoV-2 infection and were subjected to chest CT, blood collection, and nasal swab. Of these, 200 individuals were excluded, 60 owing to inconclusive RT-PCR, 60 owing to image artifacts on the chest CT, and 80 owing to incomplete filling of the medical record. A total of 259 patients were finally included in the study, 211 with a confirmed diagnosis of COVID-19 by RT-PCR and 48 with a negative diagnosis of COVID-19 by RT-PCR (Figure 1).

The study groups were homogeneous in relation to the sex of the individuals (male: Group 1, *n* = 124/Group 2, *n* = 22, *p* = 0.1 and female: Group 1, *n* = 87/Group 2, *n* = 26, *p* = 0.1), age (Group 1: 51.8 ± 17.9/Group 2: 47.6 ± 18.6, *p* = 0.14) and age groups (until 59 years old: Group 1, *n* = 140/Group 2, *n* = 35, *p* = 0.4 and 60 years-old or more: Group 1, *n* = 71/Group 2, *n* = 13, *p* = 0.4).

The clinical evaluation showed that there was no statistically significant difference regarding the time of symptom onset in the two groups [Group 1: 8.7 ± 2.8/ Group 2: 9.1 ± 1.9, *p* = 0.2]. However, most individuals were treated at the emergency room between 6 and 10 days after the onset of symptoms. The most common symptoms were cough [Group 1, *n* = 199 (94%)/Group 2, *n* = 46 (95%), *p* = 1], fever [Group 1, *n* = 154 (72%)/Group 2, *n* = 28 (58%), *p* = 0.05], myalgia [Group 1, *n* = 172 (81%)/Group 2, *n* = 38 (79%), *p* = 0.83], dyspnoea [Group 1, *n* = 169 (80%)/Group 2, *n* = 37 (77%), *p* = 0.69], headache [Group 1, *n* = 163(77%)/Group 2, *n* = 32 (66%), *p* = 0.13], and anosmia [Group 1, *n* = 154 (73%)/Group 2, *n* = 29 (60%), *p* = 0.11]. Fever shows a trend of association to Group 1, and ageusia occurred only among individuals in Group 1.

Regarding comorbidities, prevalence of diabetes mellitus and systemic arterial hypertension was similar in both the groups, but obesity was more frequent in Group 1 [Group 1, *n* = 48 (22%)/Group 2 *n* = 2 (4%), *p* = 0.00]. Peripheral oxygen saturation levels below 93% were also more frequent in this group [Group 1, *n* = 115 (54%)/Group 2, *n* = 18 (37%), *p* = 0.03], as well as minor lymphocite levels at initial attendance [Group 1: 1,344 ± 578/Group 2: 1,932 ± 405, *p* < 0.01], leukopenia (leucocytes level <4,000/mm^3^) [Group 1, *n* = 56 (26%)/Group 2, *n* = 4 (8%), *p* = 0.00], higher levels of C-reactive protein [Group 1, 64 ± 29/Group 2 51 ± 24, *p* < 0.01], and hospital admission [Group 1, 124 (58.7%)/Group 2, 19 (39.5%), *p* = 0.02] (Table 2).

**Table 2:** Epidemiological, clinical, and laboratorial characteristics of study participants (Belém, Pará, Brazil, 2020).

	**Group 1** ***n*** **= 211 (81.4%)**	**Group 2** ***n*** **= 48 (19.6%)**	**Total** ***n*** **= 259 (100%)**	***p*** **-value**
**Sex of participants**				
Male	124 (58.7%)	22 (45.8%)	146 (56.3%)	0.1
Female	87 (48.7%)	26 (54.2%)	113 (43.7%)	
Age (mean ± sd)	51.8 ± 17.9	47.6 ± 18.6	51 ± 18.1	0.14
Symptoms time in days, (mean ± sd)	8.7 ± 2.8	9.1 ± 1.9	8.8 ± 2.6	0.2
**Symptoms time in days**				
Until 5	26 (12.3%)	0 (0%)	26 (10%)	0.00^*^
6–10	132 (62.6%)	38 (79%)	170 (65%)	0.00^*^
≥1	53 (25.1%)	10 (21%)	63 (25%)	0.00^*^
**Symptoms**				
Cough, *n* (%)	199 (94%)	46 (95%)	245 (94%)	1
Fever, *n* (%)	154 (72%)	28 (58%)	182 (70%)	0.05
Myalgia, *n* (%)	172 (81%)	38 (79%)	210 (81%)	0.83
Dyspnoea, *n* (%)	169 (80%)	37 (77%)	206 (79%)	0.69
Headache, *n* (%)	163 (77%)	32 (66%)	195 (75%)	0.13
Anosmia, *n* (%)	154 (73%)	29 (60%)	183 (70%)	0.11
Odynophagy, *n* (%)	139 (65%)	28 (58%)	167 (64%)	0.40
Runny nose, *n* (%)	52 (24%)	9 (19%)	61 (23%)	0.45
Diarrhea, *n* (%)	16 (7%)	2 (4%)	18 (6%)	0.54
Abdominal pain, *n* (%)	30 (14%)	2 (4%)	32 (12%)	0.055
Ageusia, *n* (%)	24 (11%)	0 (0%)	24 (9%)	0.01^*^
**Comorbities**				
DM, *n* (%)	30 (14%)	4 (8%)	34 (13%)	0.34
SAH, *n* (%)	45 (21%)	7 (14%)	52 (20%)	0.32
Obesity, *n* (%)	48 (22%)	2 (4%)	50 (19%)	0.00^*^
SpO2 ≤ 93%, *n* (%)	115 (54%)	18 (37%)	133 (52%)	0.03^*^
**Leukocytes at initial attendance**				
<4,000/(mm3), *n* (%)	56 (26%)	4 (8%)	60 (23%)	0.00^*^
4,000–10,000/(mm3), *n* (%)	58 (27%)	27 (56%)	85 (33%)	0.00^*^
>10,000/(mm3), *n* (%)	97 (47%)	17 (35%)	114 (44%)	0.20
Lymphocytes at initial attendance (mm3), mean ± sd	1,344 ± 578	1,932 ± 405	1,453 ± 578	<0.01#
C-reative protein at initial attendance/ (mg/dL), mean ± sd	64 ± 29	51 ± 24	62 ± 29	<0.01#
Hospital admission	124 (58.7%)	19 (39.5%)	143 (55.2%)	0.02^*^
Nursery, *n* (%)	71 (33%)	11 (23%)	82 (31%)	0.17
ICU, *n* (%)	53 (25%)	8 (16%)	62 (23%)	0.26

*Group 1, COVID-19 positive; Group 2, COVID-19 negative; DM, diabetes mellitus; SAH, systemic arterial hypertension; SpO_2_, peripheral oxygen saturation; PCR, C-reactive protein; ICU, intensive care unit*.

# *ANOVA (p < 0.05)*.

* *Fisher exact test (p < 0.05)*.

**Figure 2:**
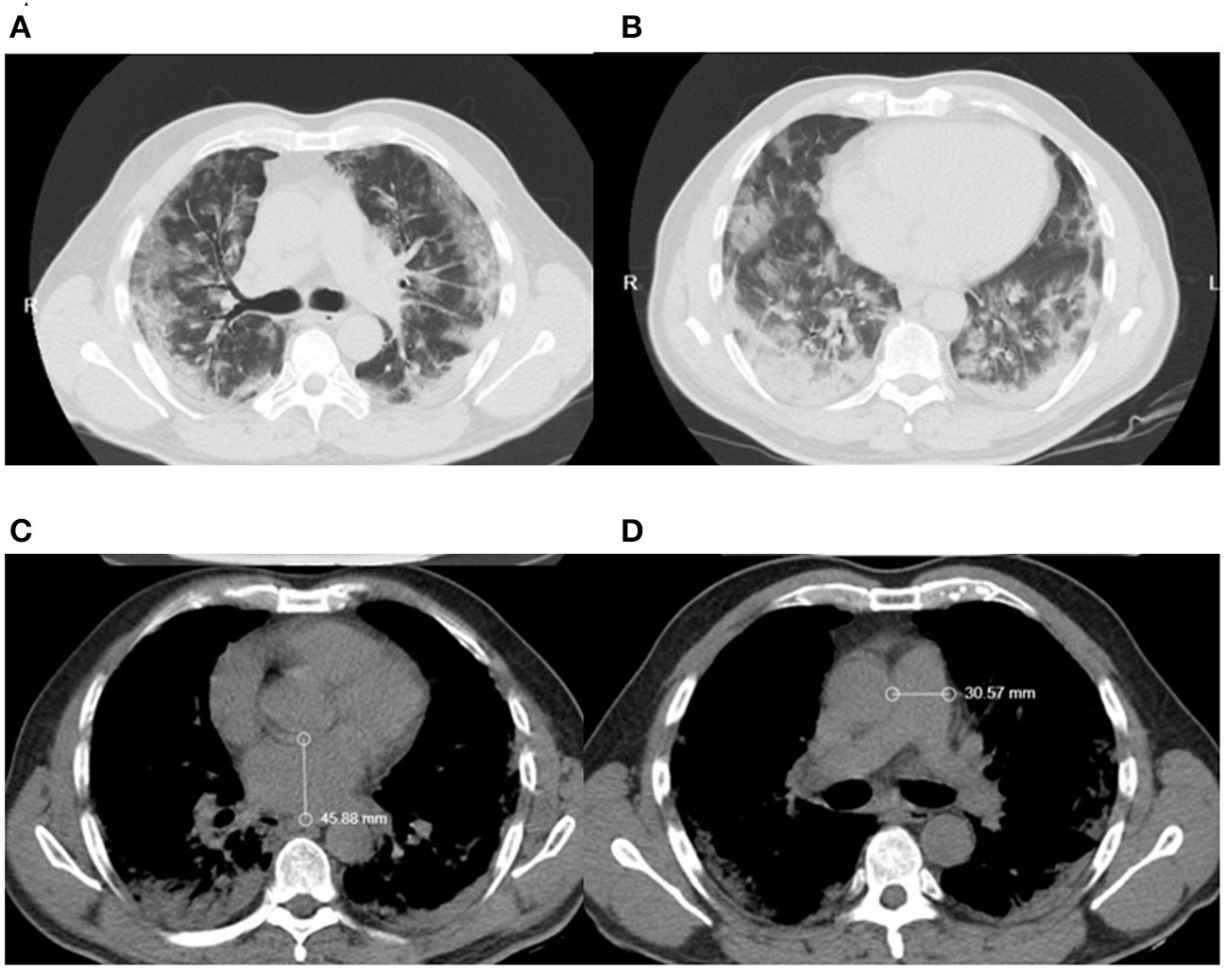
Patients chest CT showing **(A)** vascular enhancement sign **(B)** ground-glass opacities **(C)** left atrium diameter **(D)** pulmonary artery trunk diameter.

The interobserver concordance between the two radiologists who analyzed the chest CT images was 93% (*k* = 0.9304) and was determined by a kappa test. The main pulmonary findings on chest CT were ground-glass opacity [Group 1: 175 (83%)/Group 2: 24 (50%), *p* = 0.00], vascular enhancement sign [Group 1: 128 (60%)/Group 2: 15 (31%), *p* = 0.00], septal thickening [Group 1: 99 (47%)/Group 2: 13 (27%), *p* = 0.01], crazy-paving pattern [Group 1: 98 (46%)/ Group 2: 13 (27%), *p* = 0.01], and consolidations [Group 1: 92 (43%)/Group 2: 8 (16%), *p* = 0.00], all being more frequent among individuals in Group 1 (Table 3). Individuals in Group 1 also presented with a higher frequency of bilateral [Group 1: 150 (71%)/Group 2: 23 (48%), *p* = 0.00] and lower lobe injuries [Group 1: 143 (67%)/Group 2: 20 (41%), *p* = 0.00], as well as the involvement of more than 50% of the lung parenchyma [Group 1: 60 (28%)/Group 2: 7 (14%), *p* = 0.00] when compared to individuals in Group 2. There was the presence of other radiological findings, left atrium hypertrophy (Left atrium diameter > 40 mm) [Group 1: 65 (31%)/Group 2: 10 (21%), *p* = 0.21], increase in the pulmonary artery trunk diameter (diameter > 29 mm) [Group 1: 104 (49%)/Group 2: 20 (41%), *p* = 0.42] (Figure 2), and reduction in the density of the liver parenchyma (<45 UH) [Group 1: 122 (57%)/Group 2: 22 (46%), *p* = 0.14), but none showed any statistically significant difference between the two groups (Table 3).

**Table 3:** Main findings at Chest CT in symptomatic individuals by group (Belém, PA, Brazil-2020).

	**Group 1** **(*n* = 211)**	**Group 2** **(*n* = 48)**	**Total** **(*n* = 259)**	***p*** **-value**
**Pulmonary findings**				
Ground-glass opacity, *n* (%)	175 (83%)	24 (50%)	199 (76%)	0.00^*^
VES, *n* (%)	128 (60%)	15 (31%)	143 (55%)	0.00^*^
Septal thickening, *n* (%)	99 (47%)	13 (27%)	112 (43%)	0.01^*^
Crazy-paving pattern, *n* (%)	98 (46%)	13 (27%)	111 (42%)	0.01^*^
Consolidation, *n* (%)	92 (43%)	8 (16%)	100 (38%)	0.00^*^
Parenchimal bands, *n* (%)	62 (29%)	8 (16%)	70 (27%)	0.07
**Distribution of injuries**				
Bilateral injuries, *n* (%)	150 (71%)	23 (48%)	173 (66%)	0.00^*^
Lower lobe injuries, *n* (%)	143 (67%)	20 (41%)	163 (63%)	0.00^*^
Opacities <25%, *n* (%)	76 (36%)	30 (62%)	106 (40%)	0.00^*^
Opacities 25–50%, *n* (%)	75 (35%)	11 (23%)	86 (33%)	0.00^*^
Opacities > 50%, *n* (%)	60 (28%)	7 (14%)	67 (27%)	0.00^*^
**Other radiological findings**				
Left Atrium > 40 mm, *n* (%)	65 (31%)	10 (21%)	75 (28%)	0.21
PAT diameter, mean ± sd	28.5 ± 5.2	26.8 ± 5.3	28.1 ± 5.2	0.06
PAT diameter ≥ 29 mm, *n* (%)	104 (49%)	20 (41%)	124 (47%)	0.42
Hepatic parenchyma density ≤ 45 UH, *n* (%)	122 (57%)	22 (46%)	144 (59%)	0.14

*Group 1, COVID-19 positive; Group 2, COVID-19 negative; CO-RADS, The Coronavirus disease 2019 (COVID-19) Reporting and Data System; UH, Unidade Hounsfield; CT, computed tomography; VES, vascular enhancement sign; PAT, pulmonary arterial trunk*.

* *Fisher exact test (p < 0.05)*.

Opacities^*^: *findings as ground-glass opacity, consolidation, and crazy paving pattern*.

**Figure 3:**
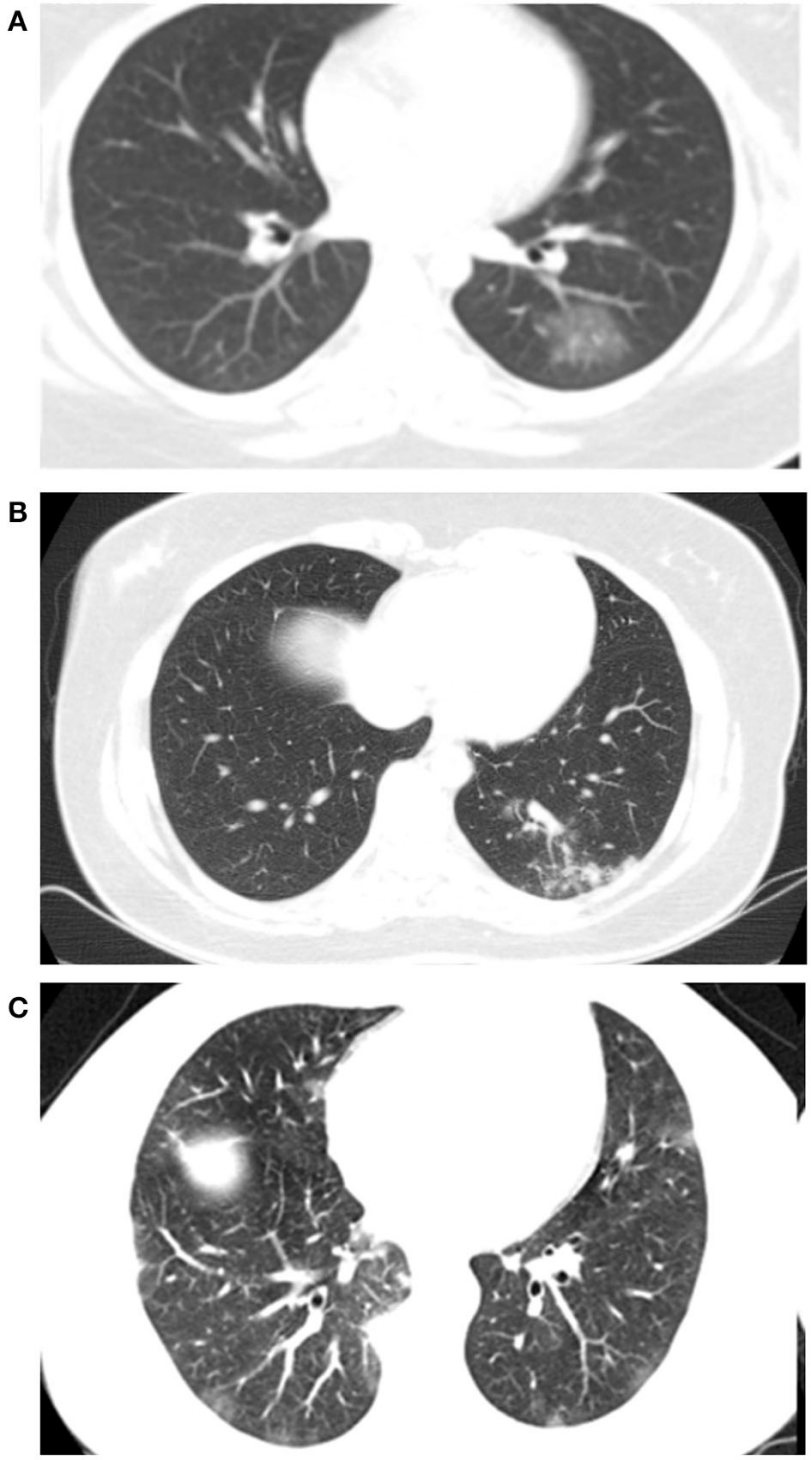
Patients chest CT showing coronavirus disease 2019 reporting and data system **(A)** CORADS 3. **(B)** CORADS 4. **(C)** CORADS 5.

Chest CT scans of each patient were analyzed based on the description of the parenchymal injuries and classified into a CO-RADS category (Figure 3).

There was a relationship between the CO-RADS category on chest CT and the results of the RT-PCR diagnostic tests for SARS-CoV-2, with a higher proportion of individuals with CO-RADS categories 4 and 5 in Group 1 [Group 1: 163(77.25)/Group 2: 24(50), *p* = 0.00] (Table 4).

**Table 4:** CO-RADS of chest CT in symptomatic individuals by group (Belém, PA, Brazil-2020).

	**Group 1** **(*n* = 211)**	**Group 2** **(*n* = 48)**	**Total** **(*n* = 259)**	***p*** **-value**
CO-RADS 4 ou 5, *n* (%)	163 (77.25)	24 (50)	187 (72.20)	0.00^*^
CO-RADS 3, *n* (%)	16 (7.58)	0 (0)	16 (6.17)	0.04^*^
CO-RADS 1, *n* (%)	32 (15.16)	24 (50)	56 (21.62)	0.00^*^
Total	211	48	259	

*CT, computed tomography; Group 1, COVID-19 positive; Group 2, COVID-19 negative; CO-RADS, The Coronavirus disease 2019 Reporting and Data System; CO-RADS 4, 5, high, very high suspicious for COVID-19; CO-RADS 3, equivocal or unsure; CO-RADS 1, very low or normal*.

* *Fisher exact test (p < 0.05)*.

## Discussion

The individuals evaluated in this study were in the acute phase of the disease with a predominance of respiratory symptoms, such as cough, fever, myalgia, dyspnoea, and headache. There was no predominance of individuals with associated comorbidities, diabetes mellitus, or systemic arterial hypertension. Changes in other systems, in this initial assessment, seem to have no observable repercussions on imaging examinations. The individuals in Group 1 had a higher incidence of imaging findings compared to those in Group 2, and the main findings on chest CT were ground-glass opacity, vascular enhancement sign, and septal thickening. The presence of CT scans classified as CO-RADS 4 and CO-RADS 5 was significantly higher in Group 1, so was the presence of a higher percentage of parenchyma involvement.

Comorbidities such as diabetes mellitus and systemic arterial hypertension have been reported to be associated with a higher probability for the development of severe forms of COVID-19 and SARS (23); however, there was no difference in the proportion of individuals with comorbidities between the groups in this study. The search for hospital care was initiated after the 6th day of symptoms by 90% of the study subjects, and the most severe respiratory symptoms of COVID-19, such as dyspnoea and hypoxemia, were noted to start on the 7th day of infection. In a study involving 138 patients, 20% of the individuals developed SARS within 8 days after the onset of symptoms and 12.3% required invasive mechanical ventilation (24). Another study reported that of the 201 patients hospitalized with COVID-19 in Wuhan, 41% developed acute respiratory distress syndrome (23).

COVID-19 has flu-like characteristics and symptoms and the most common symptoms in individuals with COVID-19-related pneumonia were fever, cough, expectoration, and myalgia. Less common symptoms were headache, dyspnoea, abdominal pain/diarrhea, pharyngeal discomfort, and chest pain (4, 11). Associated with these symptoms, many individuals also complained of loss of smell and taste, denominated as anosmia and ageusia, respectively (25–27). Lung injuries were predominantly bilateral and in the lower lobes. Ground-glass opacity in the peripheral areas is the characteristic pattern of COVID-19 (28) and is also characterized by being symmetrical and basal (5, 8, 24, 29–31).

These findings tend to change according to the stage of the disease. In the first 4 days, ground-glass opacities are the most common (76.5%); between 10 and 14 days of illness, crazy-paving pattern is the most common (62.7%); between 15 and 21 days, consolidation (75%) is commonly noted; between 22 and 28 days, linear opacities (83.1%) are seen; and in individuals over 28 days, the most common findings are ground-glass opacities [98.1%; (32)]. The pulmonary manifestations of COVID-19 can be lasting, with the presence of sequelae and residual lesions in a significant portion of the survivors (33).

Computed tomography has great sensitivity for detecting patterns related to COVID-19, but a low specificity therefore is recommended to be used in combination with a more specific diagnostic method (34–36). In a study on 1,014 patients in Wuhan who underwent RT-PCR and chest CT for COVID-19 assessment, a positive COVID-19 chest CT had a sensitivity of 97% using RT-PCR as a reference; however, the specificity was only 25% (14). CT, despite not being a completely reliable diagnostic tool, is useful in determining the severity of COVID-19 in clinical practice (37).

CO-RADS and other protocols were created by radiological societies around the world within the scope of the COVID-19 pandemic, such as the protocol created by Radiological Society of North America, both of which classify pulmonary involvement as typical, indeterminate, atypical, or negative (12, 38) and are comparable with each other in sensitivity and reliability (12, 39, 40). CT reports usually also include the estimate of pulmonary involvement, reported in percentage (41). This degree of involvement is often useful in determining severity and estimating the prognosis (37, 42). Chest CT is an important auxiliary tool in the diagnosis and acts as an indicator of the severity of pulmonary involvement in COVID-19 (43).

Computed tomography alone does not provide a reliable diagnostic confirmation. Multimodality imaging assessment in patients with COVID-19 has been shown to be useful to assess cardiac complications in this population (44). CT has the advantages of rapid application and high image resolution and can be used, among other things, for the evaluation of cardiac chambers (45–47). The findings of pulmonary artery trunk diameter with dimensions above 29 mm and left atrium hypertrophy are suggestive of cardiovascular affection, despite this, there was no statistical difference in comparison to Group 2.

As it is a systemic inflammatory disease, COVID-19 affects, among others, the gastrointestinal system (48). Individuals who developed the severe form of the disease had pathological tissue changes in the liver parenchyma, developing liver cirrhosis and non-alcoholic liver steatosis (49). An increased liver parenchyma density is suggestive of hepatic steatosis and was observed in 57% of individuals in Group 1. However, this also did not show a statistically significant difference in relation to Group 2.

The early diagnosis of COVID-19 is essential for better management of the patient, be it the decision to carry out more detailed monitoring in moderate forms, to ensure social isolation, and to prevent the spread of the disease. The gold standard diagnostic methods have high sensitivity and specificity; however, they have a turn-around time of at least 7 days.

This study reinforces the importance of CT as a rapid diagnostic adjunctive method for COVID-19. Its use allows for better decision-making by the health team, indicating the best measures to be taken according to the clinical picture and tomographic patterns of each patient, as well as, determining with greater sensitivity the suspected cases of COVID-19, leading to greater assertiveness in its handling.

Future studies must focus on the follow-up of individuals who have recovered from COVID-19 to help determine the relationship between the sequelae of the infection and imaging patterns observed in various health services. We highlight that the main limitations of this study was the small sample size and the fact that this group of patients represents those seen in only one health service; therefore, the results can possibly not be generalized to the city's population.

## Data Availability Statement

The original contributions presented in the study are included in the article/supplementary material, further inquiries can be directed to the corresponding author/s.

## Ethics Statement

The studies involving human participants were reviewed and approved by Research Ethics Committee from Hospital Universitário João de Barros Barreto (Protocol n. 4.010.595). A consent form for data use was obtained from the hospital where the participants were treated.

## Author Contributions

WV, KF, LF, JQ, and RS contributed to conception and design of the study. AD and AF organized the database, figures, and performed the statistical analysis. WV and KF wrote the first draft of the manuscript. All authors contributed to manuscript revision, read, and approved the submitted version.

## Conflict of Interest

The authors declare that the research was conducted in the absence of any commercial or financial relationships that could be construed as a potential conflict of interest.

## Publisher's Note

All claims expressed in this article are solely those of the authors and do not necessarily represent those of their affiliated organizations, or those of the publisher, the editors and the reviewers. Any product that may be evaluated in this article, or claim that may be made by its manufacturer, is not guaranteed or endorsed by the publisher.
